# On Some Symptoms of Cerebellar Tumours
1Received for publication, March 28th.


**Published:** 1905-06

**Authors:** J. Michell Clarke

**Affiliations:** Professor of Pathology, University College, Bristol; Physician to the Bristol General Hospital


"*Scire est nescire, nisi id me*
*Scire alius sciret.*"

JUNE, 1905.

### ON SOME SYMPTOMS OF CEREBELLAR TUMOURS.[1]

BY

J. MICHELL CLARKE, M.A., M.D.Cantab., F.R.C.P.,

*Professor of Pathology, University College, Bristol;*
*Physician to the Bristol General Hospital.*

THE symptoms of cerebellar tumour to which I wish first to allude are those connected with disturbances of hearing. Affection of hearing not unfrequently occurs in cerebellar tumours, because they exert pressure upon the auditory tracts or nerves. It generally takes the form of impairment of hearing only, and is either unilateral or is more marked on the side of lesion. Associated with deafness may be tinnitus aurium, especially, according to Ferrier,[2] in connection with tumours of the middle lobe. It is not common, however, to get absolute and complete deafness as in the two following cases, in which it was also accompanied by very distressing tinnitus aurium. In these cases, moreover, the deafness and tinnitus long preceded

[1] Received for publication, March 28th.

[2] Allbutt's *System of Medicine*, vol. vii., 1899, p. 378.

the pathognomonic symptoms of cerebellar tumour, and it was on account of deafness that the patient first sought advice. By the time, however, that they came under my own observation there was in the first case commencing optic neuritis in one eye, and in the second inco-ordination of movement, symptoms incompatible with disease limited to the organs of hearing, but until these signs appeared the diagnosis must have been impossible.

CASE I.—The first patient, a man æt. 40, a clerk, I saw, through the kindness of Dr. Barclay J. Baron, in May, 1897. He was well nourished and healthy looking, but grey, and looked older than his years. He had never had syphilis nor any previous illness, except rheumatic fever at the age of 14. He complained chiefly and bitterly of noises in the ears—a constant roaring, a bell ringing, or gongs striking; these first came about six years ago, and have been constant ever since. They were worse in a noise of any description, even talking increase them. They did not prevent sleep. He was stone deaf. Deafness came on in the right ear six years previously, and at that time the Eustachian tube was blocked. Dr. Baron tells me that he then treated him for double labyrinthine deafness with injections of pilocarpine. The left ear had gradually become deaf during the last six months. The noises in the ears preceded the deafness. He also complained of a feeling of uncertainty or unsteadiness of his legs. On this account he was nervous about going up or downstairs; the right leg was the weaker and more uncertain. He had never fallen, and had not suffered from giddiness.

On examination he appeared to walk naturally and briskly; there was slight swaying on standing with the eyes shut, but he could walk both forwards and backwards with his eyes closed. There was no paralysis of any cranial nerve. Swallowing normal. Speech rather rapid and monotonous, but natural. Pupils, right contracted and fixed; left dilated. They acted well to light, to accommodation, and consensually. There was fine horizontal nystagmus of both eyes in movements to either canthus, but not when at rest. There was some myopia. Right optic disc normal, left showed commencing neuritis. The visual fields, taste, and smell, were normal. No affection of sensation in any part of the body; no paralysis of the limbs or back; no muscular wasting; musculature poorly developed; electrical reactions normal. No rigidity. Knee-jerks present, but very defective, especially on the left side. Superficial reflexes normal. There was a fine constant tremor, not increased by movement, of arms and hands. Twitchings of the muscles of the legs occurred frequently, especially in the quadriceps extensor femoris. Micturition and defæcation normal.

No tenderness over head or spine, and the latter showed no deformity. Mental powers normal; memory good. He is able to go on with his work.

November, 1897. When seen again condition unchanged, except that tinnitus was worse. He could walk twelve to fourteen miles, never fell, but occasionally felt uncertain on his legs; he was not giddy.

October, 1898. Condition rather worse; sight failing, especially in left eye; he feels generally weaker, and is more easily tired; he cannot walk so well as formerly, and has difficulty in walking in the dark and in keeping steady; he walks like one intoxicated. He is now slightly giddy, and has frontal and occipital headache. No vomiting. No ataxy of movement. There is a little difficulty in starting micturition. Right pupil small and fixed, left normal and active. Nystagmus same; no oculomotor or other cranial nerve paralysis. Visual fields normal. Marked double optic neuritis; most advanced on the left side. He had now an uncertain titubating (cerebellar) gait in walking backwards or with the eyes shut, but hardly noticeable in ordinary walking. No ataxy of arms or hands. Fine tremor of legs. Both knee-jerks absent.

In November, 1898, it was noted that his gait was decidedly ataxic, and he had a tendency to walk or fall to the right. He was slow and awkward in rising from a chair. The right leg seemed weaker than the left. The right grasp by dynamometer, 30; left, 37 kilos.

At the beginning of 1899 he saw Dr. Ferrier, who agreed with the diagnosis of cerebellar tumour. He stayed two months in the Queen Square Hospital, where Sir V. Horsley did a craniotomy, removing a large piece of bone low down on the right side below the level of the tentorium and of the occipital ridge.

This resulted in improvement of general symptoms, but in March, 1899, it was noted that he had a marked cerebellar gait, much worse when his eyes were closed, and he was not able to walk without a stick. Optic neuritis about the same in the left, better in the right eye. He suffered from attacks of temporary blindness, followed by intense headache and giddiness. Nystagmus was now most marked on looking to left. Other symptoms remained as before. A considerable soft, fluctuating, painless swelling about the size of half an orange had developed over the site of the trephine opening. After this date I saw him occasionally from time to time. The general course of his illness may be given very briefly. He remained stone deaf; the noises in his ears also troubled him, but were less than at first. The headache also improved. The optic neuritis cleared up, leaving a slightly atrophic condition of left disc but fairly good sight, so that he could see to read with suitable glasses for his myopia. The ataxy of the legs continued, and to it was gradually added loss of power, with spasticity of the limbs.

He remained in this state until 1904, when he began to develop signs of bulbar paralysis from pressure, and died of this from failure of respiration with filling of the lungs in September, 1904. Leave was obtained to examine the brain, but this could not be done until forty-eight hours after death. The cerebral convolutions were flattened, and the ventricles much distended and filled with cerebro-spinal fluid. Otherwise the cerebrum was normal. The cerebellum was very much softened, and was occupied by a large, firm tumour the size of a small apple; this was oval in shape, and involved the right lateral lobe chiefly and very extensively, but also extended into the middle and left lateral lobes. From the condition of the parts the exact origin and relations of the growth could not be determined. The pons and medulla were flattened from pressure. The growth proved on microscopic examination to be a fibroma.

Thomas J., æt. 50. This patient was first seen in September, 1896, when he stated that he had enjoyed good health until two years previously. He had been quite temperate for twelve years. He had never before this illness suffered from headache, deafness, nor ear-discharge. The onset began with "a cold" two years previously, when he became absolutely deaf in the left ear; shortly afterwards his hearing began to fail in the right ear, until now he can only occasionally hear when one shouts loudly into his right ear. Soon after the deafness appeared he began to suffer very much from tinnitus aurium and giddiness. Twelve months previously his gait became staggering, "like a drunken man," and this was much worse in the dark. About this time he had a sort of epileptiform fit.

On admission he complained much of deafness and tinnitus aurium; mentally he was quite sound. Pupils equal; act well to light and accommodation. Slight nystagmus in all positions of the eyeballs, most marked in the horizontal. No cranial nerve-palsy. Speech normal. No optic neuritis. Knee-jerks were increased. There was no ankle-clonus. There was a marked "cerebellar" gait, and Romberg's symptom was present. He was treated with hypodermic injections of pilocarpin without benefit.

He left the hospital, and was seen next in May, 1898. In addition to the other symptoms, he had now dragged his left foot for over one year, and consequently had difficulty in going upstairs. Giddiness, tinnitus, deafness, and unsteadiness of gait were unchanged. He had especial difficulty in turning. Although he said the left knee felt the weaker, and inclined to give way, he tended to walk and to fall towards the right; on closing his eyes he fell backwards at once. There was some rigidity in the leg, with slight ankle-clonus; both knee-jerks were now much exaggerated, the left most. Plantar reflexes active. Double optic neuritis was present, most marked in the right eye. Slight horizontal nystagmus was seen. No cranial nerve-palsy. There was left hemianæsthesia, especially to pain, less marked over the face and head than over the body and limbs. Otherwise sensation normal. Intelligence good; he understood what was said to him by reading the movements of the speaker's lips. He complained of severe frontal headache.

In August, 1898, the staggering character of the gait and the tendency to fall to the right had increased. There was marked ataxy of movement in the left arm and hand, and, in addition and distinct from inco-ordination, a tremor of moderate amplitude, which was decidedly increased on voluntary movement. The deep reflexes were now also exaggerated in the left upper extremity.

During September and October his condition varied, but on the whole tended to become worse. The gait was now, owing to the rigidity, which was, however, never extreme, and weakness of the left leg, a compound between a cerebellar and an hemiplegic gait, and he had increasing difficulty in getting about. He had more headache, and was becoming more emotional. The optic neuritis remained at about the same intensity. The right pupil was larger than the left. The nystagmus and other symptoms were about the same.

I lost sight of this patient subsequently, and have not been able to trace him. I think, however, that the similarity in the symptoms and the course of the illness in his case makes it almost certain that he was suffering from a similar lesion to that in the first case. Both cases show the importance of bearing in mind the occurrence of deafness and tinnitus as occasional early signs of a cerebellar tumour. Fortunately, in the majority of cases of tumour in this situation we are aided by the presence of optic neuritis, which, as a rule, appears early, and soon becomes intense.

There is one other situation in which a tumour may cause complete deafness, and that is when it grows in the corpora quadrigemina; indeed, it is stated[1] that the immediate cause of deafness due to a cerebellar growth may be pressure upon the posterior corpora quadrigemina and internal geniculate bodies, through which the central auditory tract ascends.

The symptoms and course of illness in both these patients and the histological characters of the growth in Case I. corre-

[1] Dercum, quoted by Ferrier (*loc. cit.*).

spond to the description given by Fraenkel, Hunt, Woolsey, and Elsberg[1] of neuro-fibromata of the auditory nerve. It was unfortunate that in the latter case the condition of the parts was not sufficiently good to enable the exact origin of the tumour to be ascertained. The authors consider that these growths give rise to certain symptoms in order, and run such a course that they can be thereby distinguished from other tumours of the cerebellum or in the posterior fossa. These characters are:—

1. The long duration of symptoms, three to six years; whilst the *average* duration of all cerebellar tumours is two years.

2. The initial and most striking or special symptoms, namely, tinnitus aurium, Ménière's syndrome, and deafness, are referable to the auditory nerve, and are of long-standing and gradual onset.

3. The general and focal symptoms are those present in any tumour of the posterior fossa, and come on gradually in the course of the illness, the former comprising headache, vertigo, vomiting, and optic neuritis; and the latter cerebellar ataxia, homolateral or crossed paralysis of the extremities, paralysis of the cranial nerves (especially of the seventh, sixth, and fifth), nystagmus, dysarthria, dysphagia, and paralysis of the conjugate movements of the eyes.

With this description the two cases given above fairly correspond, the peculiar points being the long course, the complete deafness, with tinnitus aurium, and the appearance of these symptoms at the onset, to be followed, after a considerable interval of time, by others characteristic of cerebellar disease. The successful result of the craniotomy in the first case is also worthy of remark. The patient was relieved of headache, and the optic neuritis subsided; so that he preserved his sight to the extent that he could read quite small print, and lived in comparative comfort for more than four years after the operation.

Attacks of auditory vertigo, or the set of symptoms known as Menière's syndrome, are well known to occasionally occur in

[1] *Ann. Surg.*, 1904, xl. 293.

cases of cerebellar tumour. They may or may not be associated with deafness, and consist of severe attacks of vertigo, with tinnitus, ending in vomiting. If, as sometimes happens, these attacks are the first signs of a cerebellar tumour, preceding, as they sometimes do, all other symptoms for some little time, they may lead to a mistaken diagnosis of labyrinthine disease. Fortunately, as said above, optic neuritis occurs early in the majority of cases of cerebellar growths, and its presence would of course show that the condition present was not merely due to disease of the internal ear.

In a man, aged 45, the first symptoms of illness consisted of violent attacks of giddiness, in which he had to lie down to avoid falling, ending in vomiting, faintness, and palpitation. There was no deafness at first, and very little tinnitus. For some months these were the only symptoms, except that there was slight optic neuritis, most marked in the right eye. The presence of this optic neuritis gave the clue to the right diagnosis; without it the attacks would have been attributed to auditory vertigo. Later a staggering gait, weakness of limbs, deafness, intense optic neuritis, vomiting, headache, and mental weakness appeared, and he died about 18 months after the first onset of symptoms.

*Postmortem.*—A large fibroma was found in the left lateral lobe of cerebellum.

Optic neuritis, from its usually early occurrence so valuable in diagnosis, may, however, be absent even in large tumours of the cerebellum. Unless the other symptoms are pronounced, its absence may render the diagnosis of the nature of the cerebellar disease uncertain. Ferrier[1] states that optic neuritis was present in nine out of ten cases under his own observation, and in sixty-four which he analysed it was present in forty-six, absent in four, and not mentioned in fourteen.

In a case recently under my care optic neuritis was absent throughout, although the disease ran a very short course, only lasting five months from the first appearance of symptoms, and the tumour was a very large one. Moreover, there was a second growth in the frontal region of the cerebral hemisphere. The growth was a round-celled sarcoma, and contained large cysts. It has been stated that gliomata and sarcomata of the brain, which form cysts, are for some reason less likely to give

[1] *Loc. cit.*

rise to optic neuritis. The history of this patient is briefly as follows:—

He was a gardener, aged 44, and had always enjoyed good health until this illness. His father died of "cancer in the chest," his mother, aged 70, is weak-minded, and one sister is in an asylum for dementia. In September, 1904, he began to feel weak, and complained of numbness in the right foot, of weakness in the right arm, of attacks of vomiting, sometimes after meals, sometimes irrespective of them, and of giddiness, which had caused him to fall down several times, always towards the left side. Subsequently the giddiness increased, and was especially bad on movement; he developed a marked staggering gait, and became feeble and uncertain on his legs. The deep reflexes were increased, the plantar were flexor in type. There was no oculomotor paralysis, the pupils reacted sluggishly, the optic discs were normal; no deafness; there was no affection of sensation or of muscular sense. He became drowsy, and took to his bed a fortnight before he died in a state of coma, which supervened gradually. During the last ten days of his life he lay coiled up in bed on his right side, and if moved from that position returned to it again.

*Postmortem.*—A large soft sarcoma of loose structure was found to occupy the central portion of the middle lobe of the cerebellum, and to extend for a considerable way into each lateral lobe, replacing the greater part of the white matter. A cystic tumour, the size of a walnut, was also found in the frontal lobe at the posterior end of the first frontal convolution, and there were other tumours at the root of the right lung, and in the left supra-renal body.

The other symptom to which I wish to refer is tremor. The cerebellum is one part of the brain in which a tumour may give rise to a tremor of "intentional" character. This was so in the following case:—

Wm. B., aged 3 years. The illness began with sickness and diarrhœa, which lasted a week; after this it was found that he could not stand, but was just able to hobble about by helping himself with his arms. The child screamed a great deal at times as if in much pain.

On admission he looked healthy and well nourished. The head was very large, measuring 22½ inches in circumference, the anterior fontanelle and the sutures for some distance from it were widely dilated. The child could not stand, and when sat up in bed he tended to fall backwards. When lying down he could move his legs about freely, except that he had some difficulty in raising them off the bed. The deep reflexes were present, the superficial absent, except the plantar, which showed Babinski's sign. The pupils were dilated, and did not act to light or accommodation. There was no oculomotor paralysis, and when at rest the face appeared fairly symmetrical, but when he laughed or cried the right side of the face remained motionless, and the mouth was slightly drawn over to the left; the right side of the face was also but less noticeably paralysed for voluntary movements. There was a marked tremor of the arms and hands on movement, most evident on the right side, and this tremor was somewhat wide in range; when the limbs were lying at rest there was no tremor, but the least movement brought it out. The tremor closely resembled that of disseminate sclerosis in its "intentional" character. The child's speech was somewhat drawling; he was quite intelligent, and not deaf. There was very intense optic neuritis in both eyes.

During the further course of the illness the legs became rigid, and the deep reflexes were increased. There was no complete facial paralysis other than above described. The head gradually increased in size. The right upper extremity became very rigid, and he ceased to use it, grasping objects with his left hand only.

He lay on his back in a natural posture, but during the last two weeks of his life the arms were generally in a peculiar position, the right was rigidly adducted to the side, extended at the elbow and wrist with the fingers flexed, while the left was abducted, the forearm raised above the shoulder, and the fingers free. There was no rigidity in the left arm, and the tremor on movement continued throughout, but it was noticeable that as the rigidity increased in the right arm the tremor grew less marked and finally disappeared. He gradually became comatose and died.

*Postmortem.*—There was a condition of pronounced hydrocephalus due to enormous distension of the ventricles. The grey and white matter of the hemispheres were greatly reduced in thickness. The cause of this distension was found to be a very large tumour growing from the under surface of the cerebellum, which had infiltrated the cerebellum, practically replacing the middle and extending into each lateral lobe. The pons and medulla were much flattened and thinned out from pressure. The structure of the growth was that of a glioma.

The interesting feature of this case, apart from its exceptionally rapid course, was the peculiar tremor, which resembled that of disseminate sclerosis, in being absent when the limb was at rest and supported, and becoming very evident on movement.

Tremors of this kind are sometimes found in tumour of the brain in three positions, most commonly when they occur in the region of the optic thalamus or in the mid-brain; less often when the growth is in the corpora quadrigemina or cerebellum. Other characters of the tremor are its increase on emotional or other psychical excitation, its absence during sleep, the affection of the upper extremity only, at any rate for a considerable time, and predominance in the upper extremity throughout. Further, as in this case, if spastic rigidity develops in the limb in the course of the illness, the tremor diminishes or disappears.

At first I thought from this symptom that the growth would be found in the optic thalamus in the case under consideration, especially as at first sight he appeared to have another peculiar symptom ascribed to growths in this situation, *i.e.* unilateral loss of emotional, not voluntary, facial movements. Later, however, it was seen, though the child's age made it difficult to determine, that both kinds of facial movements were affected, and the signs of cerebellar disease became clear.

In a former paper[1] I reported two cases in which this tremor was associated with tumours of the optic thalamus. In one of these there was degeneration in the superior peduncle of the cerebellum, and to a lesion of these structures, Professor Ferrier, from his experiments on monkeys, is inclined to refer the tremor in these cases.

In a recent paper[2] Dr. Gordon Holmes brings forward a large body of evidence to show that these tremors are particularly associated with lesions of the mid-brain, and that they are directly due to involvement of the nucleus ruber or of some part of the rubro-spinal system; that is to say, of the tract which by means of the superior cerebellar peduncle connects the opposite lobe of the cerebellum with the nucleus ruber, and by the rubro-spinal tract connects this nucleus, by fibres passing downwards, with the opposite side of the cord. The clinical symptoms of Dr. Gordon Holmes's cases, and the *post-mortem* results in those in which an autopsy was made, indicated involvement of either the superior cerebellar peduncle or the nucleus ruber. The anterior end of this nucleus projects, as he points out, within the usual anatomical

[1] *Brit. M. J.*, 1891, i. 1,283; 1901, ii. 1,406.

[2] Brain, *Autumn*, 1904, xxvii. 327.

limits of the thalamus, with which it has connections; so that it would be involved in growths of the posterior end of the thalamus of any size, and thus the relative frequency of the tremor in growths affecting the thalamus would be explained.

### THE CAUSES AND TREATMENT OF BALDNESS.

